# Untargeted metabolic profiling reveals geography as the strongest predictor of metabolic phenotypes of a cosmopolitan weed

**DOI:** 10.1002/ece3.4195

**Published:** 2018-06-22

**Authors:** Natalie Iwanycki Ahlstrand, Nicoline Havskov Reghev, Bo Markussen, Hans Christian Bruun Hansen, Finnur F. Eiriksson, Margrét Thorsteinsdóttir, Nina Rønsted, Christopher J. Barnes

**Affiliations:** ^1^ Natural History Museum of Denmark University of Copenhagen Copenhagen Denmark; ^2^ Department of Mathematical Sciences University of Copenhagen Copenhagen Denmark; ^3^ Department of Plant and Environmental Sciences University of Copenhagen Copenhagen Denmark; ^4^ ArcticMass Reykjavik Iceland

**Keywords:** cosmopolitan weed, environmental conditions, geographic location, geographic patterns, herbivory, local adaptation, metabolic phenotype, phenotypic plasticity, soil pH

## Abstract

Plants produce a multitude of metabolites that contribute to their fitness and survival and play a role in local adaptation to environmental conditions. The effects of environmental variation are particularly well studied within the genus *Plantago*; however, previous studies have largely focused on targeting specific metabolites. Studies exploring metabolome‐wide changes are lacking, and the effects of natural environmental variation and herbivory on the metabolomes of plants growing *in situ* remain unknown. An untargeted metabolomic approach using ultra‐high‐performance liquid chromatography–mass spectrometry, coupled with variation partitioning, general linear mixed modeling, and network analysis was used to detect differences in metabolic phenotypes of *Plantago major* in fifteen natural populations across Denmark. Geographic region, distance, habitat type, phenological stage, soil parameters, light levels, and leaf area were investigated for their relative contributions to explaining differences in foliar metabolomes. Herbivory effects were further investigated by comparing metabolomes from damaged and undamaged leaves from each plant. Geographic region explained the greatest number of significant metabolic differences. Soil pH had the second largest effect, followed by habitat and leaf area, while phenological stage had no effect. No evidence of the induction of metabolic features was found between leaves damaged by herbivores compared to undamaged leaves on the same plant. Differences in metabolic phenotypes explained by geographic factors are attributed to genotypic variation and/or unmeasured environmental factors that differ at the regional level in Denmark. A small number of specialized features in the metabolome may be involved in facilitating the success of a widespread species such as *Plantago major* into such wide range of environmental conditions, although overall resilience in the metabolome was found in response to environmental parameters tested. Untargeted metabolomic approaches have great potential to improve our understanding of how specialized plant metabolites respond to environmental change and assist in adaptation to local conditions.

## 1 INTRODUCTION

Plants produce a multitude of specialized metabolites that contribute to their fitness and survival, and play a role in their ability to adapt to local environmental conditions. Specialized metabolites play a role in defense against herbivores and pathogens (Bowers & Stamp, 1992), provide protection from harmful ultra‐violet (UV) radiation (Murai, Takemura, Takeda, Kitajima, & Iwashina, 2009), and are essential for fostering mutualistic relationships with other organisms such as pollinators and mycorrhizal fungi (Schweiger, Baier, Persicke, & Muller, 2014). Specialized metabolites also are the source of many of our plant‐based medicines and therefore have value to human health and well‐being (Briskin, 2000). Much attention has therefore been placed on environmental and geographic factors influencing specialized metabolite production in plants, particularly in crop species and in model plant systems (Agrawal, Conner, Johnson, & Wallsgrove, 2002; Asai, Matsukawa, & Kajiyamal, 2016; Carrari et al., 2006; Dan et al., 2016; Hirai et al., 2004; Lasky et al., 2012; Tarczynski, Jensen, & Bohnert, 1993; Riedelsheimer et al., 2012). However, the importance of natural variation in the environment in explaining metabolite variation within plant species remains little understood (Maldonado et al., 2017; Moore, Andrew, Külheim, & Foley, 2014).

Out of the circa 250 species that comprise the genus *Plantago* (Rahn, 1996; Rønsted, Chase, Albach, & Bello, 2002), the specialized chemistry of two species, *Plantago lanceolata* L. and *P. major*, has been the focus of a vast body of chemical ecology research. Due to these species’ widespread and nearly global distributions in nature, their medicinal value (Samuelsen, 2000), and because both can be easily grown under controlled conditions, *P. lanceolata* and *P. major* L. serve as excellent model organisms to explore biochemical responses and improve understanding of local adaptation (Fuchs & Bowers, 2004; Pankoke, Buschmann, & Mueller, 2013; Sutter & Muller, 2011).

A wide array of environmental factors have been shown to influence the types or concentrations of specialized metabolites produced by the model *Plantago* species, including plant/leaf age and phenology (Barton, 2008; Hanley et al., 2013; Pankoke et al., 2013; Sutter & Muller, 2011), herbivore and pest damage (Bowers, Collinge, Gamble, & Schmitt, 1992; Sutter & Muller, 2011), genotype (Adler, Schmitt, & Bowers, 1995; Al‐Mamun, Abe, Kofujita, Tamura, & Sano, 2008; Barton, 2012; Bowers et al., 1992; Marak, Biere, & van Damme, 2002; Zubair et al., 2012), habitat type (Adler et al., 1995), plant competition (Barton & Bowers, 2006; Pankoke, Hopfner, Matuszak, Beyschlag, & Muller, 2015; Waschke, Hancock, Hilker, Obermaier, & Meiners, 2015), associations with microorganisms including arbuscular mycorrhizal fungi (Bennett & Bever, 2007; Bennett, Macrae, Moore, Caul, & Johnson, 2013; Fontana, Reichelt, Hempel, Gershenzon, & Unsicker, 2009; Schweiger et al., 2014; Wang, Bezemer, van der Putten, & Biere, 2015), nutrient levels (Darrow & Bowers, 1999; Jarzomski, Stamp, & Bowers, 2000; Miehe‐Steier, Roscher, Reichelt, Gershenzon, & Unsicker, 2015; Pankoke et al., 2015), UV levels (McCloud & Berenbaum, 1999; Murai et al., 2009), and variation in geography and climate (Mølgaard, 1986; Murai et al., 2009; Pellissier, Roger, Bilat, & Rasmann, 2014; Reudler & Elzinga, 2015).

When investigating the complex and often interrelated effects that environmental factors have on plant metabolic phenotypes, it is becoming increasingly popular to use untargeted metabolomic approaches, as fewer *a priori* assumptions are made, allowing for the detection of metabolic responses that were overlooked using targeted approaches (Pankoke et al., 2013; Schweiger et al., 2014; Sedio, Rojas Echeverri, Boya, & Wright, 2017; Sutter & Muller, 2011). Despite the extensive chemical ecology literature that exists for *Plantago*, still little is understood about metabolome‐wide responses (but see Sutter & Muller, 2011; Pankoke et al., 2013, 2015; and Schweiger et al., 2014). As is the case for most model plant systems, the regulation and expression of plant metabolites have traditionally been assessed under controlled conditions and focus on the response of a handful of targeted (specific) metabolites to simplified environmental factors. In *Plantago*, it is the iridoid glycosides aucubin and catapol, and the caffeoyl phenylethanoid glycosides verbascoside and plantamajoside, that have been most widely studied as chemical response variables, particularly due to their antiherbivore and medicinal activity (Bowers et al., 1992; Mølgaard, 1986; Reudler et al., 2013; Rønsted, Franzyk, Mølgaard, Jaroszewski, & Jensen, 2003).

There is a growing need to investigate how varying environmental conditions affect plant metabolomes in situ. The majority of metabolite investigations, even for the well‐studied *Plantago* species, have been conducted on plants grown under controlled conditions, and therefore, much of the environmental complexity is hidden given the simplicity of controlled conditions. Variation in environmental factors tested under controlled conditions may vary at levels not occurring under natural conditions and may not reflect responses that are shaped by multiple interacting factors found *in situ* (Maldonado et al., 2017). Given that metabolic phenotypes are a product of the interaction of a number of factors, including genetic and environmental factors, a plant's metabolome can be regarded as the ultimate response of biological systems (Fiehn, 2002; Lopez‐Alvarez et al., 2017). Thus, screening the metabolomes of locally adapted plants that have been exposed to natural environmental conditions throughout their lifetimes is an essential step in improving our understanding of the role that a plant's metabolome plays in local adaptation to environmental conditions. Significant advances in the field of metabolomics, specifically with today's high‐throughput analytical instruments and bioinformatic tools (Lankadurai, Nagato, & Simpson, 2013; Sedio et al., 2017), now allow us to more thoroughly explore the influence of environmental factors on the variation in metabolic phenotypes using complex multivariate data.

The aim of this study was to use an untargeted metabolomic approach to investigate intraspecific variation in metabolic features and disentangle the relative effects of environmental and geographic factors measured at different spatial scales on the metabolome using the widespread weed, *Plantago major*, as a model. Highly plastic species such as *P. major*, with large geographic ranges, have the ability to exhibit higher intraspecific variation in physiology and morphology and serve as good models to study local and regional adaptations (Soolanayakanahally, Guy, Silim, Drewes, & Schroeder, 2009). We therefore investigate the extent to which environmental factors such as differences in habitat type, light levels, phenological stage, leaf area, and soil conditions are important drivers of metabolic phenotypes of plants sampled *in situ* from 15 populations across Denmark. Given the complexity of assessing the effects of environmental factors measured under natural conditions, we first use variation partitioning to allocate variation of these explanatory variables on the foliar metabolomes, based on methanolic extracts. We subsequently applied a novel and highly conservative mixed linear modeling approach to identify statistically significant metabolic features associated with environmental and geographic variation. We further apply a networking analysis on co‐occurring metabolites to identify patterns in metabolic phenotypes across Denmark. Lastly, we test whether the induction of nonvolatile, polar metabolites can be detected in response to localized leaf damage caused by herbivory in situ, by comparing undamaged and damaged leaves collected from the same individuals.

## 2 MATERIALS AND METHODS

### 2.1 Plant population sampling

*Plantago major* is a short‐lived perennial herb belonging to the genus *Plantago* (Plantaginaceae family). *Plantago major* was selected for this study because of its widespread distribution, its ability to grow in a wider range of natural and semi‐natural conditions compared to its congener, *P. lanceolata*, and the presence of previous studies using Danish and Dutch populations which provides background information (Haeck, van der Aart, Dorenbosch, Van der Maarel, & Van Tongeren, 1982; Mølgaard, 1986; Kuiper & Bos, 1992). In addition to its ability to adapt to a wide range of habitat types and environmental conditions, *P. major* exhibits high phenotypic plasticity and can vary extensively in morphology based on growth conditions, even within populations (Mølgaard, 1986; Samuelsen, 2000; Warwick & Briggs, 1980). Two subspecies are recognized in Denmark, *P. major* subsp. *major*, and *P. major* subsp. *intermedia* (Gilib.) Lange (syn. *P. major* subsp. *pleiosperma* Pilg.); we restricted sampling to the former more widely distributed subspecies in this study because past studies have shown secondary chemistry and other phenotypic traits to differ between subspecies (Mølgaard, 1986). Plants are self‐fertile, wind pollinated, but nonrhizomatous, and although hundreds of individuals can persist in a small area, low genetic diversity is expected within populations (van Dijk & van Delden, 1981; Mølgaard, 1986; Rahn, 1996;).

**Figure 1:**
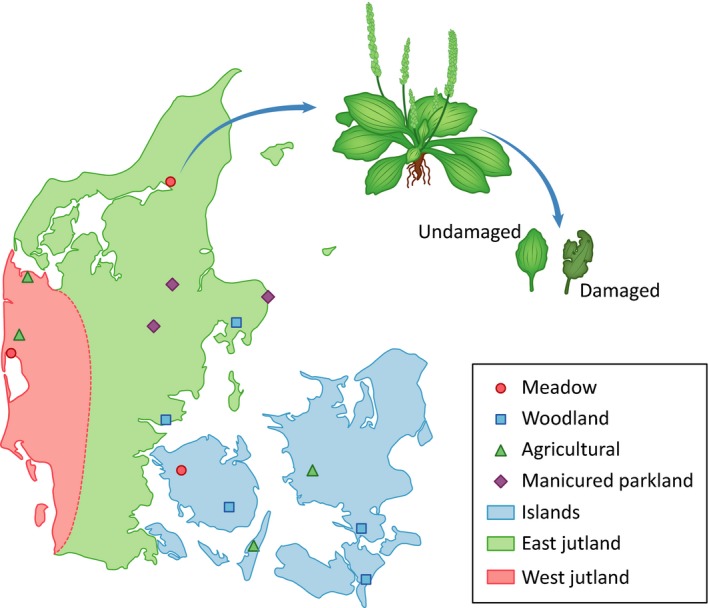
Map of 15 populations of *Plantago major* sampled across Denmark from three geographic regions, western Jutland, eastern Jutland, and the islands. Sampling was conducted in meadow, woodland, agricultural, and manicured parkland habitats. Three individuals of the species were sampled at each population, and two leaves (one showing localized herbivore damage and one undamaged) were collected from each individual

Fifteen naturally occurring populations of *Plantago major* were selected across Denmark to capture a range of different habitat types (woodland, meadow, agricultural, and parkland), geographic regions and geological conditions known from the various islands and mainland in Denmark (Table 1). Given the low expected genetic diversity between individuals of *P. major* at a population level in Denmark, we selected three plants to serve as biological replicates in our population‐level sampling. Two fully expanded leaves of similar age (based on their position in the rosette) and similar size were sampled from each plant; one leaf that showed no visible signs of herbivore damage (“undamaged”), and one leaf that did (“damaged”). This resulted in a paired dataset consisting of an undamaged and damaged leaf from each plant individual. Young leaves (i.e., those that had not fully unfurled) as well as older leaves (i.e., those showing changes in coloration or senescence) were avoided in our sampling. In total, 90 leaves (45 undamaged leaves and 45 damaged) were collected from 45 plants in the 15 different natural populations (Figure 1). In order to minimize effects caused by temporal variation, field sampling was conducted over a span of 5 days, in July 2015. Photographs were taken of each plant, and the phenological stage was recorded (vegetative, immature flowers, or flowering). Meta‐data is presented as Supplementary Information, Table S1. In addition, one herbarium voucher was collected from each population and deposited at Herbarium C at the Natural History Museum of Denmark in Copenhagen. Environmental conditions (qualitative light level and soil samples), as well as associated species, were recorded for each population (Table 1 and Figure 1).

**Table 1:** Populations of *Plantago major* L. sampled across Denmark. Vouchers are deposited in Herbarium C

Population name	Location	Latitude	Longitude	Geographic region	Habitat type	Light levels	Voucher No.
Aalborg	Nørresundby, Jutland	57.08	9.91	Eastern Jutland	meadow	full sun	NI578
Falster	Halskov, Falster	54.80	12.09	Islands	forest	shade	NI570
Grenaa	Grenaa, Jutland	56.41	10.92	Eastern Jutland	manicured park	full sun	NI580
Hannerupskov	Fredericia, Jutland	55.59	9.72	Eastern Jutland	forest	shade	NI582
Langeland	Rudkøbing, Langeland	54.92	10.71	Islands	agricultural	full sun	NI571
Nørre Nissum	Nissum Seminarieby, Jutland	56.55	8.42	Western Jutland	agricultural	full sun	NI576
Nyråd	Nyråd, Zealand	55.01	11.96	Islands	forest	part shade	NI569
Øjesø	Søttrup, Jutland	56.29	10.61	Eastern Jutland	forest	part shade	NI581
Randers	Randers, Jutland	56.47	10.02	Eastern Jutland	manicured park	full sun	NI579
Ringkøbing	Ringkøbing, Jutland	56.10	8.23	Western Jutland	meadow	full sun	NI574
Ringkøbing Ejstrup	Ringkøbing, Jutland	56.18	8.28	Western Jutland	agricultural	full sun	NI575
Silkeborg	Silkeborg, Jutland	56.23	9.67	Eastern Jutland	manicured park	full sun	NI577
Slæbæk	Slæbæk, Fyn	55.11	10.57	Islands	forest	shade	NI572
Slagelse	Slagelse, Zealand	55.43	11.46	Islands	agricultural	full sun	NI573
Vissenbjerg	Vissenbjerg, Fyn	55.38	10.13	Islands	meadow	part shade	NI573

### 2.2 Leaf area and localized damage by herbivory assessment

To assess the role that leaf area and localized damage caused by herbivory have on the expression of metabolic features within an individual plant, photographs of undamaged and damaged leaves from each plant (*n* = 90) were taken on grid paper to give scale in absolute and analyzed with ImageJ software (National Institute of Mental Health, Bethesda, Maryland, USA), following similar methods to Pellissier et al. (2014). Total leaf area, (*A*_L_), and the area missing due to herbivore damage (*A*_D_) were calculated. Damage (%) caused by herbivory for each leaf was calculated as *A*_D_/*A*_L_*100%. Leaf damage in the majority of the *P. major* populations sampled was caused by invertebrate herbivores (slugs and caterpillars) based on observed feeding patterns on the leaves and the presence of these herbivores observed in the field (although invertebrates were not collected or identified). This is consistent with observations made by Mølgaard (1986) in Denmark. Leaves from each plant were put into 50 ml tubes and stored on ice while in the field, then put into storage at −20°C until processing.

### 2.3 Analyses of soil samples

To explore variation in edaphic conditions within and between populations, three soil samples were taken at each sampling location, each collected adjacently to the three plants sampled, using stainless steel soil sampling rings (100 cm^3^ in volume, Eijkelkamp, the Netherlands). Soil samples were loosely covered and air‐dried until completely dry, before being stored in airtight containers. Soil samples (*n* = 45) were analyzed for pH, electrical conductivity (EC), and carbon to nitrogen ratio (C/N). Samples were gently ground using a mortar and pestle and passed through a 2‐mm sieve. To measure pH, 10 g of sieved soil was transferred to a 50 mL tube and 25 ml of distilled water was added (1:2.5 w/v). Tubes were shaken for 1 min and then every 10 min for 50 min. pH was measured in the upper part of the colloid suspension with a Metrohm 914 pH/Conductometer (Metrohm, Switzerland), calibrated beforehand using three buffers (pH 4, 7, and 9). To measure EC, five grams of sieved soil was dispersed in 25 ml of distilled water (1:5 w/v). Tubes were shaken every 10 min for an hour, then let to rest for 10 min. EC was measured in the upper layer using a Metrohm 914 pH/Conductometer after calibration with one conductivity standard (Reagecon TM, NIST 20 μS/cm, Fisher Scientific, USA). For C/N, a 15 g subsample of sieved soil was ground to a fine powder using a custom‐designed ball mill. One‐hundred milligrams of milled soil was combined with the same weight of tungsten. Soil C/N was measured by Dumas combustion on a Vario Macro Cube element analyzer (Elementar Analysensysteme GmbH, Hanau, Germany). Sulfanilamide was used as a standard in three different weights, and acetanilide was used to calibrate drift after every 15 samples.

### 2.4 Spatial variables describing geographic variation

A Euclidean distance matrix was created between sampling locations using the vegdist function the *R* package “vegan” (Oksanen et al., 2017). Geographic variation between populations was then incorporated into downstream analyses by constructing principal components of neighborhood matrices (PCNM) using the pcnm function, also in “vegan,” and PCNM one and two are used as explanatory variables to describe spatial structure and are referred to as geographic distance herein (Borcard & Legendre, 2002).

In addition, three broad geographic regions in Denmark were designated to investigate geographic effects at a higher scale. These include: (a) western Jutland, a zone that represents the part of the land base that was not affected by the last Weichsel glacial maxima in Eurasia (Svendsen et al., 2004), (b) eastern Jutland, and (c) the islands. Both eastern Jutland and the islands are dominated by young soils formed on moranic tills, while major parts of western Jutland are an outwash plain dominated by sandy soils and weathered deposits from previous glaciations (European Soil Bureau, 2005; Jakobsen, Hermansen, & Tougaard, 2015; Krüger, 1987).

### 2.5 Chemical extraction

Variation in metabolic phenotypes was investigated on a subset of the metabolome based on methanol extracts from *Plantago major* leaves. Methanolic extractions are effective in extracting the specialized metabolites such as iridoid glycosides, caffeoyl phenylethanoid glycosides, and other nonvolatile, polar metabolites (Pankoke et al., 2013; Rønsted, Göbel, Franzyk, Jensen, & Olsen, 2000; Rønsted et al., 2003). Fresh leaf samples (*n* = 90) were stored in 50 ml tubes at −20°C until processed. Leaves were then freeze‐dried using a ScanVac CoolSafe (Labgene, Denmark), and dried leaf tissue for each sample was transferred to 2 ml Eppendorf tubes (Sigma‐Aldrich, USA) filled with glass beads (2 mm diameter), and ground and homogenized using a TylerLyser (Qiagen, USA). A 30 mg subsample of the ground material was weighed out for each sample, transferred to 1.5 ml Eppendorf tubes, and 1,200 μL of 80% HPLC‐grade methanol (Sigma‐Aldrich, USA) was added (40 μl/mg dried tissue). Tubes were incubated at 70°C for 10 min (with gentle agitation), followed by centrifuging (5 min, 14,000 rpm, Thermo Fisher, USA). 1,000 μL of each supernatant was pipetted into new tubes and the resulting solutions were dehydrated with a ScanSpeed MaxiVac Evaporator (Labogene, Denmark) for 12 hr at 35°C, 1,200 rpm. Dehydrated samples were stored at −18°C until further analyses. Samples were resuspended using 1 mL of 99.9% HPLC‐grade methanol (Sigma‐Aldrich, USA), and put into an ultrasonic water bath for 10 min. Resuspended solutions (0.5 mL) were filtered using 0.21 μm MC Centrifugal Filter Units (Ultrafree^®^, Merck Millipore), and centrifuged for 5 min at 8,000 rpm (16,000 *g*). The filtered extracts were transferred to Waters LC certified glass vials (Waters corp., Milford, MA, USA) before undergoing untargeted metabolomic analyses using ultra‐high performance liquid chromatography coupled with time of flight mass spectrometry (UPLC‐QToF‐MS).

### 2.6 UPLC‐QToF mass spectrometry

UPLC‐QToF‐MS were performed with a Waters Acquity UPLC system, coupled to a Waters Synapt G1 mass spectrometer equipped with an electrospray ionization (ESI) probe. The analytical column used was an ACQUITY UPLC HSS T3 C18 (2.1 mm × 100 mm i.d.; 1.8 μm) (Waters corp., Milford, MA, USA), maintained at 35°C. The gradient system mobile phase consisted of buffer A: 0.1% formic acid, B: acetonitrile +0.1% formic acid (all mobile phase solvents were CHROMASOLV^™^ for HPLC ≥99.9%, Sigma‐Aldrich), at a flow rate of 0.5 mL/min. The injection volume of 2 μl was followed by a gradient hold for 0.8 min starting at 98% mobile phase A, then a linear gradient from 98% A to 80% A in 1.7 min followed by a linear gradient to 5% A for 2.5 min. The gradient was held for 1.0 min before going back to the initial conditions and equilibrated for 1.8 min. The total chromatographic run time was 8.0 min. The sample manager temperature was maintained at 4.0°C. The capillary voltage was 3.2 kV, the cone voltage was set to 35 V and extraction cone 5.3 V. The scan time was 0.1 s in the mass range of 100–1,000 Dalton. Source temperature was 125°C, and desolvation temperature was 450°C, at a flow rate of 800 L/hr (N_2_) and cone gas flow rate 50 L/hr. Leucine encephalin was used as reference lock mass calibrant. Data acquisition was carried out using MassLynx 4.1 software (Waters corp., Milford, MA, USA), with data stored in centroid mode.

The instrument was calibrated prior to analyses following manufacturers recommendations. A standard of verbascoside, 10 μg/μl (primary pharmaceutical reference standard, Sigma‐Aldrich), was injected four times to ensure the instrument was performing consistently across days and batches. The 90 leaf extracts were split in two separate blocks and were run in triplicate over 2 days. Samples were randomized within each block, so that triplicate 1 was ran on the first day, triplicate 3 on the second, and the second of the triplicates split between days 1 and 2. A quality control (QC) consisting of a pooled 10 μL aliquot of every sample was injected ten times at the start of each block to condition the column, and the QC was injected three times after injecting every 20 samples. A total of 135 samples were injected (45 samples, run in triplicate), and 70 QCs, in each batch.

### 2.7 UPLC‐QToF‐MS data preprocessing and filtering

Peak data were preprocessed using MarkerLynx (Waters corp., Milford, MA, USA), using the following parameters: marker intensity threshold (counts) 200, retention time tolerance 0.20, mass window 0.05, replicate % minimum 80, and deisotoping data, for the retention time period 0.35 to 6.70 min. Eight of the quality control (QC) samples injected at the start of each run were excluded from data preprocessing analyses, as they are part of the column conditioning controls. A total of 541 unique mass features were retained after preprocessing, each representing a potential biomarker. The number of mass features may be higher than the number of actual metabolites due to the occurrence of fragments and adducts. Peak intensities were normalized to the total marker intensity and exported for analyses with their respective retention times and masses. We refer to mass features as metabolic features herein, and the nomenclature used to denote each metabolite feature incorporates the retention time and specific mass for each, for example, 2.41_343.82 is a metabolic feature that has a retention time of 2.41 min and a mass of 343.82. It was not the intent of this study to identify the chemical structure of metabolic features, but to reliably “fingerprint” the foliar metabolome in a comparable manner.

Principal Component Analysis (PCA) was used to visualize the metabolic feature data and to ensure that QC samples clustered tightly, indicating good instrument performance and repeatability between and within batches. Filtering of the metabolic feature data was performed using 48 QC samples. Metabolic features were removed from the data set (and from any further analyses) if the relative standard deviation of the QC samples for that feature was >30%, following Food and Drug Administration (FDA) guidelines for biomarker discovery in untargeted liquid chromatography–mass spectrometry analyses (Dunn et al., 2011). Filtering reduced the numbers of features from 541 to 197. Means were calculated for the triplicate injections recorded for each metabolite feature.

### 2.8 Targeted analyses of known metabolites

Screening for metabolic features in a subset of the overall metabolome offers an advantage when seeking to identify changes in metabolic phenotypes in response to environmental factors. However, to test the response of metabolites known for their antiherbivory effects in *Plantago* (Rønsted et al., 2000, 2003), 10 standards were ran using the same UPLC analyses described above and a baseline for how variable these compounds are in natural populations across Denmark was established. A method file was created for analysis in the TargetLynx application of MassLynx (Waters corp., Milford, MA, USA), based on the retention times and *m*/*z* for each of the metabolites. Unfiltered metabolite feature data was processed in TargetLynx to screen for these known metabolites. The standard compounds included aucubin, verbascoside (Sigma‐Aldrich, USA), melittoside, gardoside, salidroside, arborescoside, geniposidic acid, asperuloside, ixoroside, 10‐acetoxymajoroside (isolated in previous studies by Rønsted et al., 2000, 2003; and provided for this study by Søren Rosendal Jensen, Danish Technical University). A standard for plantamajoside was unfortunately not available for this study, which is a metabolite that has previously been found to differ across Danish populations of *P. major* (Mølgaard, 1986).

### 2.9 Nonmetric multidimensional scaling

After quality filtering, a total of 197 metabolic features were retained for downstream analyses. Metabolic feature data was visualized by generating Bray–Curtis similarity measures and nonmetric multidimensional scaling models (NMDS) in the *R* package “vegan” to visualize patterns between populations, habitat types, and geographic regions (R Core Team, 2016; Oksanen et al., 2017).

### 2.10 Variation partitioning analysis of metabolic phenotypes

All 197 features were analyzed using variation partitioning (Borcard, Legendre, & Drapeau, 1992) to explore the explanatory power that the soil variables (soil pH, EC, and C/N), geographic region, and phenological stage had on metabolic phenotypes, using the varpart function within the *R* package “vegan” (R Core Team, 2016; Oksanen et al., 2017). Geographic region and distance were found to have co‐linear relationships, and therefore, distance was not included as a separate factor in variation partitioning analyses. Leaf area and localized damage [%] caused by herbivory were assessed in a separate variation partitioning model, using a reduced matrix based on differences between an undamaged and damaged leaf to account for repeated sampling of the same plants (*n* = 45). Metabolic feature intensities from damaged leaves (*M*_D_) were subtracted from the undamaged (*M*_U_) to obtain single values for each plant individual, and similarly, single values for features were calculated by subtracting the damaged leaf area (*M*_D_) feature value from the undamaged area (*M*_U_) before undergoing variation partitioning analyses.

### 2.11 Conditional log‐normal model analysis

In order to conduct a statistically valid analysis with power sufficient enough to detect significant differences in metabolic features associated with environmental and geographic variation and address low sampling numbers, we use the two‐step model proposed by Skou, Markussen, Sigsgaard, and Kollmann (2011) (see also Thiele & Markussen, 2012). The two‐step model includes a log‐normal model and a binary model and is well suited to address metabolomic data where all observations are non‐negative, but a substantial portion of the observations are zero values, as a result of many metabolic features being not present or below the detection limit. Skou et al. (2011) did not baptize their model, but for future reference we suggest that this model is called the *conditional log‐normal model*. A log‐normal model often fits well for strictly positive observations, and the conditional log‐normal model is an extension that allows for zero observations that also occur frequently in ecological datasets. The details of the conditional log‐normal model are presented as Supplementary Information, Appendix S1).

The effects of geographic region (either as western Jutland, eastern Jutland, islands), geographic distance (two continuous axes), habitat type (agricultural, forest, manicured park, meadow), light levels (full sun, part shade, shade), phenological stage (vegetative, immature flowers, or flowering), damage caused by herbivores (undamaged, damaged), leaf area and soil pH were analyzed in separate models, allowing us to identify metabolic features that differ significantly according to each of these seven explanatory variables tested. A stratified sampling design was employed to ensure that a minimum of three populations were represented in each of the categories investigated (geographic region, habitat type, and light levels). In all analyses, we corrected for leaf area (logarithmic scale) and soil pH (quadratic scale). Soil EC and C/N were found to be correlated with soil pH based on Spearman's Rank coefficient (EC, *ρ* = 0.49, *p *<* *0.001) (C/N, *ρ* = 0.39, *p *=* *0.001), and therefore, soil pH was used to represent edaphic factors in our modeling.

Conditions set out to run the models (detailed in Supplementary Information, Appendix S1) resulted in a slightly different number of metabolic features suitable for the modeling of each of the seven explanatory variables. Table 2 lists the number of features included in the analyses for each factor. The number of features tested for each feature was recorded, and Type I errors corrected to *q*‐values using the false discovery rate (FDR, Benjamini & Hochberg, 1995). The *q*‐values below 20%, 10%, and 5% were recorded, and Tukey post hoc tests were performed on all metabolic features found to have a *q*‐value of less than 20% (FDR *q*‐value < 0.20).

**Table 2:** Explanatory variables used in the conditional log‐normal model along with the number of statistically significant metabolic features detected (after FDR correction, *q*‐value < 0.20)

Factor	No. of features used in the models	No. of significant features found (*q* < 0.20)	No. significant at *p* < 0.001	No. significant at *p* < 0.01	No. significant at *p* < 0.05
Geographic region	193	30	4	12	21
Geographic distance	193	9	1	8	7
Habitat type	189	4	2	4	8
Soil pH	195	8	5	3	6
Light level	188	0	0	2	3
Phenological stage	189	0	0	0	0
Leaf area	193	3	3	3	11
Damage by herbivory	193	0	0	1	8

A Venn Diagram with the overlap in significant metabolic features identified (FDR *q*‐value < 0.20) for five of the seven environmental and geographic factors tested was constructed in the package “VennDiagram” in *R* (Chen, 2016).

### 2.12 Network analysis of co‐occurrence

Co‐occurrence networks have been implemented within community ecology using high‐throughput sequencing data, providing insight into ecological interactions between species (Barberán, Bates, Casamayor, & Fierer, 2012; Duran‐Pinedo, Paster, Teles, & Frias‐Lopez, 2011). Here we use this approach on metabolic feature data, using features that were shown to differ significantly with geographic region (30 features in total). Spearman's rank correlations were calculated in a matrix using the “vegan” package of *R* (R Core Team, 2016; Oksanen et al., 2016), with a Spearman's correlation coefficient (*ρ*) < 0.372, which was equivalent to a *p*‐value < 0.01 (Junker & Schreiber, 2008) was considered valid co‐occurrence between features. A network of features (“nodes”) and co‐occurrences (“edges”) between nodes was created with qgraph in *R* and visualized using the program Cytoscape (Shannon et al., 2003; Eskamp et al., 2012). Nodes were set as pie charts represent average feature abundance for each geographic region. The network analysis was then used to identify regionally distributed clusters of co‐occurring features, which could serve as a metabolic fingerprint.

### 2.13 Testing for differences in known antiherbivory compounds

Mann–Whitney U tests were performed in *R* on the output from TargetLynx to test for significant differences between the intensities recorded for the known antiherbivory compounds between undamaged and damaged leaf treatment groups.

## 3 RESULTS

**Figure 2:**
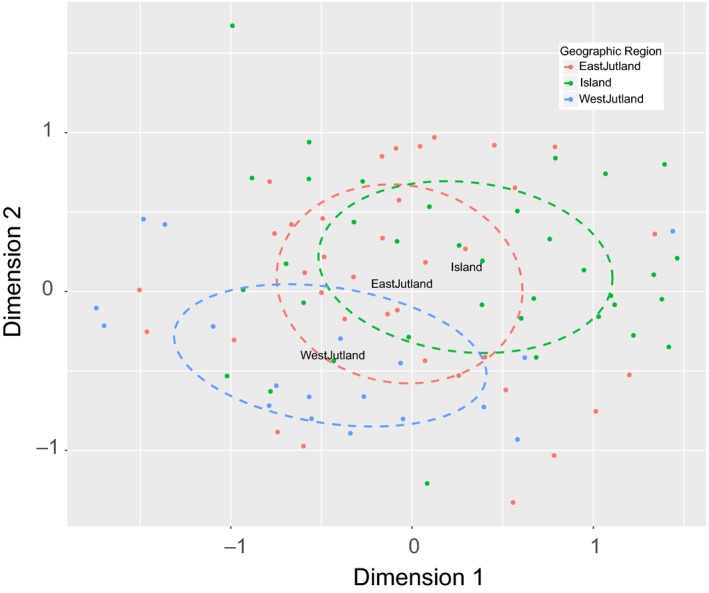
Nonmetric multidimensional scaling plot based on 197 metabolic features showing a weak geographic signal between western Jutland (red) and the islands (blue), with eastern Jutland (green) intermediate between the two. 95% confidence intervals for the three geographic regions are shown in dashed lines

After data filtering, 197 metabolite features were retained for analyses. Nonmetric multidimensional scaling (NMDS) performed on all 197 metabolite features revealed a high degree of similarity between foliar metabolomes of *P. major* collected across Denmark. No strong clustering of samples was observed according to habitat type, population (sampling site), plant identity, or leaf damage, showing an overall resilience of the metabolome; however, populations from western Jutland were more restricted in the ordination space of the NMDS plot, particularly along dimension 2 (Figure 2).

**Figure 3:**
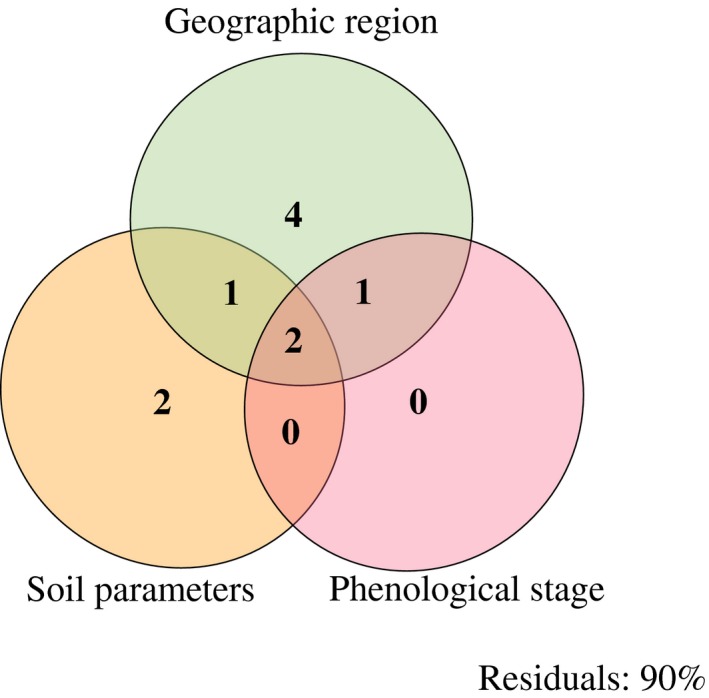
Results of the variation partition analyses for soil parameters (*F *=* *2.03, *p *=* *0.02), geographic region (*F *=* *3.74, *p *<* *0.001), and phenological stage (*F *=* *1.93, *p* = 0.04), for all 197 metabolic features

Variation partitioning was performed to quantify effects on the metabolome from soil parameters (pH, EC, C/N), geographic region, and phenological stage. Soil parameters explained 5% of the variation seen in the metabolic features, and geographic region explained 8%. There was an overlap of two percent of the variation between the three factors tested, and each of the three factors explained significant parts of the variation, soil parameters (*F *=* *2.03, *p *=* *0.02), geography (*F *=* *3.74, *p *<* *0.001), and phenological stage (*F *=* *1.93, *p* = 0.04) (Figure 3). However, a high proportion (90%) of the variation was unexplained (residuals). A second variation partitioning approach was performed on the reduced metabolic features matrix (*n* = 45) with the differences in leaf area and difference in herbivory damage (%) analyzed between undamaged and damaged leaves from each plant. While leaf area explained 9% of the variation of the metabolic features (*F *=* *4.88, *p *=* *0.009), damage had no effect (*F *=* *0.85, *p *=* *0.39), thus residuals explained 91%.

Geographic region, geographic distance, habitat type, soil parameters, light level, phenological stage, leaf area, and damage caused by herbivores were used as explanatory variables, in seven independently run analyses using the conditional log‐normal model, evaluating their relative contributions in explaining differences in metabolic features. The number of metabolic features included in the model differed slightly for each explanatory variable (Table 2), based on the criteria and conditions explained in Supplementary Information, Appendix S1. A total of 44 unique metabolic features (numbered as SF1‐44, Table 3) were found to differ significantly after FDR correction, with the majority (31 significant features) being explained by geographic region and distance (Table 2). Geographic region (as defined by three groupings: eastern Jutland, western Jutland, and the southern islands, Figure 1) was found to have the greatest magnitude of effect on differences in the metabolome; thirty of the 193 metabolic features tested in the model were found to differ statistically after FDR correction (*q*‐values < 0.20). Nine metabolic features of the 193 investigated where found to differ significantly based on geographic distance (as measured by principal coordinates of neighbor matrices), and all but one of these were shared with the features found to differ significantly in modeling of the variable geographic region.

**Table 3:** Significant metabolic features and their *q*‐values listed after FDR correction (*q*‐value < 0.2). A conditional log‐normal model was run separately for each of the seven explanatory variables tested and no significant features were found for herbivory or phenology; therefore, these variables are not included in the table

Feature ID No.	Feature (RT_*m*/*z*)	Geographic region	Geographic distance	Habitat type	Soil pH	Leaf area
SF 1	0.4123_532.8735				0.04	
SF 2	0.413_402.9161				0.19	
SF 3	0.4252_288.9357				0.04	
SF 4	0.427_174.9568				0.04	
SF 5	0.4291_434.8712				0.04	
SF 6	0.4492_258.9202				0.03	
SF 7	0.444_226.9666	0.13				
SF 8	0.4558_548.766	0.19				
SF 9	0.4566_112.9868	0.13				
SF 10	0.5182_343.1217	0.11				
SF 11	0.5295_313.1141			0.19		
SF 12	0.5269_701.1875	0.11				
SF 13	0.5327_377.0835	0.07	0.18			
SF 14	0.5336_391.1007			0.05		0.03
SF 15	0.5371_719.1995	0.05	0.19			
SF 16	0.5441_179.0569	0.13				
SF 17	0.5458_683.2223	0.18				
SF 18	0.5474_341.1072	0.09				
SF 19	0.549_387.1139	0.07				
SF 20	0.5672_677.177	0.11				
SF 21	1.7812_205.0356				0.04	
SF 22	1.9965_391.1224	0.05	0.15			
SF 23	2.0039_183.066	0.19				
SF 24	2.4247_477.159	0.05	0.009			
SF 25	2.4385_375.1298	0.00002	0.15			
SF 26	2.4401_751.2618	0.0014	0.10	0.08		
SF 27	2.6484_403.1228	0.17				
SF 28	2.9868_305.0685	0.18				
SF 29	3.0324_401.1447	0.13				
SF 30	3.2758_639.1919	0.09				
SF 31	3.3009_312.1924	0.01				
SF 32	3.4658_285.0393		0.15			
SF 33	3.3768_923.1482	0.17				
SF 34	3.3852_461.0694	0.13				
SF 35	3.459_347.1712					0.04
SF 36	3.5804_373.1835					0.03
SF 37	3.6066_475.1252	0.07	0.15			
SF 38	3.6764_607.1995	0.01	0.10			
SF 39	3.8453_285.0392			0.13		
SF 40	4.0745_299.0538	0.11				
SF 41	4.227_325.2002	0.07				
SF 42	4.7537_309.2055	0.19				
SF 43	4.9429_275.2012	0.13				
SF 44	5.1038_441.2839				0.07	

Second to the geographic factors, soil parameters had the next largest effect on differences in metabolic features (Table 2). Eight features were found to differ significantly according to pH. Four features were found to differ significantly in response to habitat type, and three metabolic features were found to differ significantly for leaf area. Light levels (i.e., whether the plants were growing in populations that had either full sun, part shade or full shade), phenological stage (vegetative, immature flowers, or flowering), and damage caused by herbivory did not explain any significant differences between metabolite features (Table 2).

**Figure 4:**
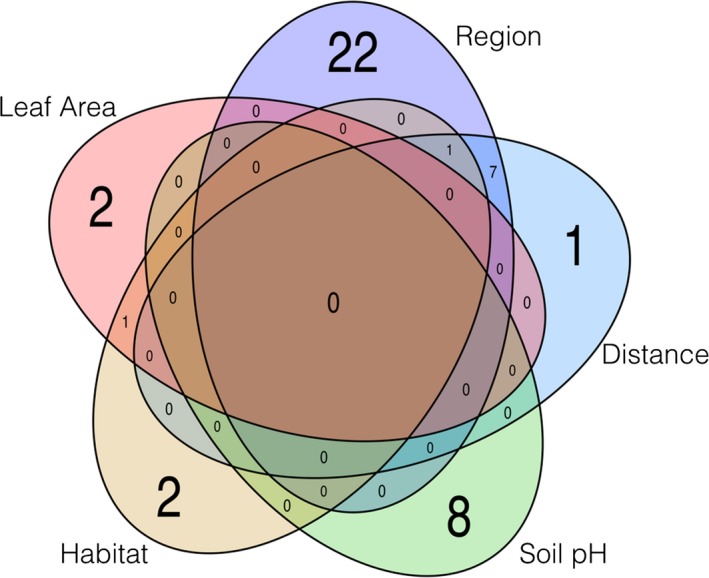
Venn diagram showing overlap in significant metabolic features identified in the conditional log‐normal modeling run separately for soil pH, geographic region, geographic distance, habitat type, and leaf area. The variables herbivory and light levels were not found to have any significant metabolic features and are therefore not shown in diagram

Despite differences detected in metabolic features in response to environmental and geographic factors, little overlap was seen in the metabolic features identified as significant under each of the explanatory variables examined, and not a single feature was found to be shared across all explanatory variables examined (Figure 4—Venn diagram; Table 3). Seven significant features were found to be shared between the two geographic factors tested, only one feature (SF14) was found to overlap between leaf area and habitat type, and one feature (SF26) was found to overlap between habitat type and the geographic factors. All eight features found to differ significantly under soil pH were not shared with any of other explanatory factors tested (Figure 4).

### 3.1 Network correlation analysis with features varying by geographic region

**Figure 5:**
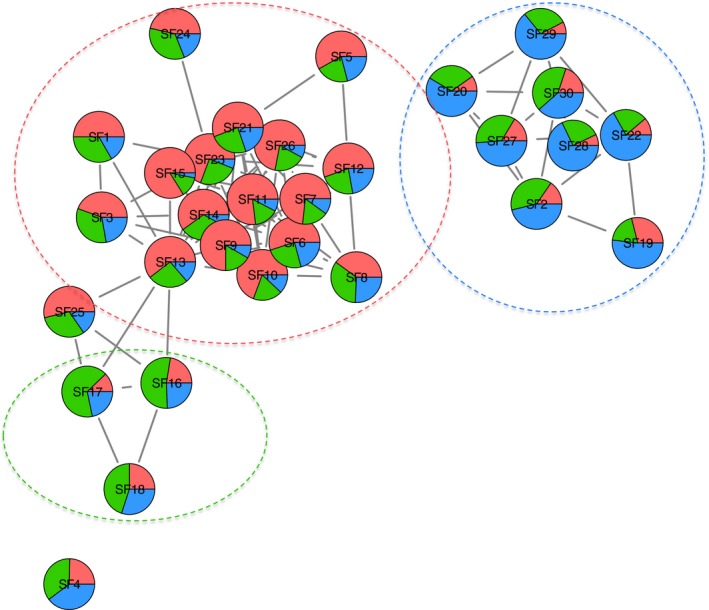
Illustration of the networking analysis with the 30 significant features (SF) identified using the conditional log‐normal model for the three geographic regions examined: Western Jutland (red), eastern Jutland (green), and islands (blue). Retention times and *m*/*z* for each SF are listed in Table 3

To investigate the degree of co‐occurrence of the 30 significant features explained by geographic region (Table 3), we undertook a network analysis based on Spearman's Rank coefficients. Taking only the positive correlations, the network analysis illustrates that strong co‐occurrence patterns exist within the significant features and that unique patterns are associated with each of the three geographic regions, particularly in western Jutland where the largest proportion of significant metabolite features were found (Figure 5).

### 3.2 Targeted analyses

Six of the ten known metabolites for which chemical standards were available (aucubin, gardoside, geniposidic acid, melittoside, verbascoside, and 10‐acetoxymajoroside) were detected in the unfiltered metabolic feature data, based on the retention times and mass spectra of these metabolites; however, the intensities of these six metabolites did not differ significantly between undamaged and damaged leaf treatment groups based on Mann–Whitney *U* tests (data not shown). Two of the known metabolites, aucubin and melittoside, were also detected in the filtered dataset of 197 metabolic features, yet were not among the 44 significant features which were identified in our conditional log‐normal model.

## 4 DISCUSSION

### 4.1 Environmental and geographic factors regulating the metabolome

We investigated variation in the foliar metabolome of *Plantago major* growing under natural environmental conditions across Denmark and identified metabolic features, beyond the traditionally studied antiherbivore metabolites, that differed in response to environmental and geographic factors, at varying spatial scales. Due to its high phenotypic plasticity and ability to adapt to a wide range of environmental conditions (Lotz & Blom, 1986; Rahn, 1996; Wolff, 1991), we hypothesized that diversity in the metabolome could be a factor involved in the plant's success and therefore expected that metabolic features would vary in response to environmental conditions such as differing habitat types, light levels, and soil conditions (Gratani, 2014). However, the limited number of metabolic features found to be regulated by environmental properties overall (i.e., only eight features for soil pH, four features for habitat type, and three features for light levels) show a relatively high innate resilience of the metabolome to changing external stimuli.

This study most notably demonstrated the importance of geographic scaling in detecting differences in metabolic phenotypes, where broad geographic regions (eastern Jutland, western Jutland, and the islands) were found to explain the highest percentage of variation of the metabolome and the largest number of individual features. Geographic factors, including distance, isolation, latitudinal, and longitudinal gradients, have previously been shown to be important in explaining intraspecific variation in targeted metabolites for vascular plants, reflecting possible adaptations to local conditions (Davey, Burrell, Woodward, & Quick, 2008; Moles et al., 2011; Züst et al., 2012). In performing networking analysis on untargeted metabolomic data, the current work also demonstrates unique patterns of co‐occurrence, such that many of the metabolite features co‐occur in plants sampled within the same geographic region. These metabolic phenotypes could be a useful approach in identifying features and metabolic pathways associated with factors such as herbivory, other environmental stressors, or genotypic diversity.

In line with our findings, variation in metabolic traits due to geographic factors has previously been associated with genotypic variation in *Plantago lanceolata* (i.e., Reudler & Elzinga, 2015), and in wild populations of *Arabidosis thaliana* (L.) Heynh. (Lasky et al., 2012), and *A. lyrata* subsp. *petraea* (L.) O'Kane & Al‐Shehbaz (Davey et al., 2008). These associations may reflect that plant genotypic variation increases with increasing geographic distance (Nybom, 2004) and highlight that there is an interdependence between genotype and metabolic phenotype (Fiehn, 2002). Significant differences found in metabolic features associated with geographic region in this study could therefore reflect upon underlying genetic variation between populations. However, in widespread weedy species such as *P. major*, with likely few barriers to its dispersal and a low expected genetic variation within populations (Verhoeven, Macel, Wolff, & Biere, 2010; Wolff & Morgan‐Richards, 1998), greater spatial distances are likely required to detect genetic variation Incorporating genetics in future studies may test this assumption.

Climatic and geological conditions, as well as biological factors including herbivore communities and pathogens are also likely to differ across the three geographic regions, thus, it cannot be ruled out that these and/or other unmeasured factors are driving the significant differences in metabolic features we observed between regions. For example, variation in local and regional herbivore communities has been shown to drive differences in metabolic profiles (Agrawal, 2011; Mølgaard, 1986; Züst et al., 2012). Similarly, climatic factors have been shown to explain variation in metabolic profiles across broad geographic regions (Laskey et al., 2012; Reudler & Elzinga, 2015). To further elucidate the effects of spatial scale from other factors, future research should include a more comprehensive suite of biotic and abiotic variables, including climate and genetic data, with a greater emphasis on quantitative data.

Although to a lesser degree, we also found natural variation in soil pH to be an important factor in explaining differences in metabolic phenotypes in *P. major*. This demonstrates that differences in edaphic conditions within a given habitat may result in differences in the expression of specialized metabolites in the metabolome, even with low population sampling numbers. Nutrient levels and nutrient availability are influenced by and often correlated with soil pH (Sims & Patrick, 1977), and changes in both pH and nutrient availability have been shown to influence the concentrations and composition of secondary metabolites produced in *Plantago* species (e.g., Darrow & Bowers, 1999; Jarzomski et al., 2000; Miehe‐Steier et al., 2015; Pankoke et al., 2015). In the current study, pH significantly accounts for changes in eight unique metabolic features, and no overlap was found with the significant metabolic features explained by geographic or other factors, which suggests these features are associated with local adaptation independent of geographic and genotypic variation (Latzel, Janecek, Dolezal, Klimesova, & Bossdorf, 2014; Metlen, Aschehoug, & Callaway, 2009).

Phenological stage, which has been well documented to influence specialized metabolites in model *Plantago* species (i.e., Barton, 2008; Hanley et al., 2013; Pankoke et al., 2013; Sutter & Muller, 2011), was not found to be an important factor in explaining metabolic variation *in situ*. Any differences due to phenological stage, were explained by differences in geographic region, which is a particularly important finding considering the difficultly in standardizing phenological stage when sampling plants growing *in situ* across great geographic distances.

### 4.2 Metabolic responses to herbivory

To the best of the authors’ knowledge, this is the first study on metabolic responses to localized herbivory, where a paired study design was adopted to investigate undamaged and damaged leaves from the same plant, growing under natural environmental conditions. Even although none of the metabolic features detected in this study were found to differ significantly between undamaged and damaged leaves, metabolites specific to herbivory could be expressed systematically (i.e., a whole plant response), or could simply have been undetectable, and we therefore cannot conclude that polar metabolites in *Plantago major* are not induced in response to herbivory. Past experiments on target metabolite induction in *Plantago* have found induction to be short‐lived and therefore difficult to detect (i.e., Bowers & Stamp, 1993; Darrow & Bowers, 1999; Fuchs & Bowers, 2004). The timing of the metabolomic screening in our study, as well as in other studies where no or insignificant differences were found (i.e., Jarzomski et al., 2000; Stamp & Bowers, 1996; Sutter & Muller, 2011), could limit detection of induced metabolites. Additional work should aim to investigate important factors such as the time and type of herbivore damage under *in situ* conditions.

### 4.3 Model validation

The statistical approach developed here allowed for the conservative testing between metabolic and environmental data that did not follow normal distributions and allowed us to address a metabolomic dataset with many zero values, which typically constrain the use of more traditional statistical analyses for ecological and biological data matrices, and further addressed issues in statistical power with low sampling numbers (Supplementary Information). Rigorous filtering steps were used in our metabolic feature data which substantially reduced the number of features available for statistical analyses. Although this conservative filtering prior to statistical analyses could result in missing important metabolic features, we believe that our conservative methods, including using FDR correction for multiple testing, improved our confidence levels in identifying significant metabolic differences and removed a high level of possible Type I error, which was particularly important in the absence of controlled experiments. The statistical approach developed here could also be useful in future studies of similar complex ecological data in other systems.

## 5 CONCLUSIONS

A surprisingly limited number of metabolic features were found to be regulated by environmental factors in this study. The greatest differences in metabolic phenotypes were detected across broad geographic regions, and this finding is largely explained by geographic separation, which in turn could reflect genotypic differentiation or variation in other unmeasured climatic and biological factors. This work also provides evidence that a small number of metabolic features are associated with local adaptation to soil pH and are independent of the effects of geography. No effects of herbivore damage were found between undamaged and damaged leaves of the same plants; however, further studies are required to investigate the induction of specialized metabolites in response to herbivory *in situ* and to characterize herbivore communities over broad geographic regions. In addition, future metabolomic studies conducted *in situ* would benefit from greater number of individuals sampled from each population, as well as controlled greenhouse studies being run in tandem, allowing for more rigorous hypothesis testing based of the patterns observed in situ.

## CONFLICT OF INTEREST

None declared.

## AUTHORS’ CONTRIBUTIONS

CJB, NIA, and NR conceptualized the study. NIA, NHR, and CJB conducted field sampling. NIA and NHR prepared samples for analyses UPLC analyses, and NIA and HCBH prepared and conducted the soil analyses. FE, and MT produced the metabolic feature data and NIA analyzed the data. BM, CJB, and NIA designed and undertook statistical analyses. NIA wrote the manuscript with CJB, BM, FE, and NR. All authors read and commented on the manuscript and approved the final version. Authors declare no conflict of interests.

## DATA ACCESSIBILITY

Metabolic feature data and environmental and geographic factor data generated for this study is available from the Dryad Digital Repository : https://doi.org/10.5061/dryad.1q0s4gf.
