# Supplementary material for: Untargeted metabolic profiling reveals geography as the strongest predictor of metabolic phenotypes of a cosmopolitan weed
Source: Ecol Evol. 2018 Jun 22;8(13):6812–26. doi: 10.1002/ece3.4195 (PMC6053570; doi:10.1002/ece3.4195)
Supplement: Supplementary file 1 [file ECE3-8-6812-s001.docx]

**Untargeted metabolic profiling reveals geography as the strongest predictor of metabolic phenotypes of a cosmopolitan weed.**

Natalie Iwanycki Ahlstrand, Nicoline Havskov Reghev, Bo Markussen, Hans Christian Bruun Hansen, Finnur F. Eiriksson, Margrét Thorsteinsdóttir, Nina Rønsted, Christopher J. Barnes.

**Supplemental Information**

Appendix S1

## Conditional log-normal model analysis

For each metabolic feature, there are 90 observations in a paired design with two observations for each of 45 plants within 15 populations. These observations all are non-negative, and have a substantial proportion of zero observations corresponding to the features being not present or below the detection limit. To make a statistically valid analysis with power sufficient enough to detect differences given low sampling numbers, we found it necessary to use the two-step model proposed by Skou et al. (2011) (see also Thiele & Markussen, 2012). To describe this statistical model, let $Y\geq0$ be the response of one of the metabolic features. Then the probability of $Y$ being strictly positive is modelled by a binary regression with *log-link*, and, conditionally on having a strictly positive observation, the logarithmic response is modelled by a normal regression, that is, we assume linear models for model parameters $\nu$and $\mu$ such that:

$$\log P\left( Y>0 \right)= \nu and for Y>0 that\log Y\sim N\left( \mu,\sigma^{2} \right).$$

Skou et al. (2011) did not baptize their model, but for future reference we suggest that this model is called the *conditional log-normal model*. A log-normal model often fits well for strictly positive observations, and the conditional log-normal model is an extension that allows for zero observations that also occur frequently in ecological datasets. A further advantage is that the logarithm of the mean response may be expressed by the model parameters via the equation:

$$\log\left( mean\left( Y \right) \right)= \log P\left( Y>0 \right)+ \log\left( mean\left( Y | Y>0 \right) \right)=\nu+\mu+ \frac{\sigma^{2}}{2}.$$

The covariance matrices for regression parameter estimates associated to $\nu$ and $\mu$ may simply be added since the binary regression on $Y>0$ vs. $Y=0$, and the normal regression on $\log Y$ for the subset of observations with $Y>0$, are done on separate datasets. Moreover, when computing contrasts between predicted means, the variance term $\sigma^{2}$ disappears. This implies that a null hypothesis of equality of predicted logarithmic means may be evaluated by *F*-tests in the conditional log normal model. This approach was used in the statistical analysis.

The effects geographic region (either as western Jutland, eastern Jutland, Islands), geographic distance (two continuous axes), habitat type (agricultural, forest, manicured park, meadow), light levels (full sun, part shade, shade), phenological stage (vegetative, immature flowers, or flowering), damage caused by herbivores (undamaged, damaged), leaf area (continuous, linear effect on logarithmic scale), soil pH (continuous, linear and quadratic effect), were analyzed in separate models for the separate metabolic features, allowing us to identify metabolic features that differ significantly according to each of the explanatory variables tested. In all analyses, we corrected for leaf area (logarithmic scale) and soil pH (quadratic scale). As an example, we describe the modeling for the analysis of the explanatory variable geographic region. Here we used the following models:

1. Binary model

$$\nu=\alpha_{\nu}\left( GeographicRegion \right)+\beta_{\nu}*\log\left( LeafArea \right)+\gamma_{\nu}*pH+\delta_{\nu}*\left( pH \right)^{2}+A_{\nu}(Plant)$$

1. Log-normal model

$$\mu_{i}=\alpha_{\mu}\left( GeographicRegion \right)+\beta_{\mu}*\log\left( LeafArea \right)+\gamma_{\mu}*pH+\delta_{\mu}*\left( pH \right)^{2}+A_{\mu}\left( Plant \right)+B_{\mu}(Population)$$

With fixed effects $\alpha_{\nu},\beta_{\nu}, \gamma_{\nu}, \delta_{\nu}$ and $\alpha_{\mu},\beta_{\mu}, \gamma_{\mu}, \delta_{\mu}$, and random effects $A_{\nu}$ and $A_{\mu},B_{\mu}$ modelling the potential correlations imposed by the experimental design. The log-normal part of the model uses plant ID and population (site) as random effects. Since the glmer function from the in *R* package ‘lme4’ used to fit the binary regression only allows for a single random effect without becoming numerically unstable, we opted to include plant ID as the random effect variable, since population is nested within plant ID. Similarly, in order to have good numerical properties, we only analyzed metabolic features that have at least two strictly positive responses for each level of geographic region, and the binary part was only included if all levels in addition also had at least one zero response. Finally, metabolic features which, despite these precautions, resulted in numerical problems in the estimation algorithms due to convergence issues, were excluded from further analysis. These conditions resulted of the 197 metabolic features being used in the modeling of geographic region, and a slightly different number of metabolic features was found to be suitable for modeling of each of the seven explanatory variables. **Table 2** lists the number of features included in the analyses for each. The *p*-value for the null hypothesis of equal predicted logarithmic means for each metabolic feature was computed. The *p*-values were then corrected in order to control the false discovery rate (FDR, Benjamini & Hochberg, 1995). In the results, we report how many metabolic features had a *p*-value computed, and how many had FDR-corrected *p*-values (risk of being false positives) below 20, 10 and 5 percent. Tukey post hoc tests were performed for all metabolic traits found to differ significantly, after FDR correction (FDR q-value < 0.20).

This procedure was repeated for the remaining six explanatory variables: geographic distance, habitat, light levels, phenological stage, soil pH, leaf area, and leaf damage caused by herbivory.

Table S1. Meta-data for *Plantago major* leaf material collected from 15 populations across Denmark. Two leaves collected from three plants at each of 15 sites.

| Plant ID | Site Name | Damage | Geography | lat | long | pH | EC | C_N | Habitat | Light | Leaf Area | Percent Damage | Phenology |
| --- | --- | --- | --- | --- | --- | --- | --- | --- | --- | --- | --- | --- | --- |
| 1 | Aalborg | Damaged | East Jutland | 57.08 | 9.91 | 5.44 | 56.65 | 11.40 | Meadow | Full Sun | 3037.16 | 1.32 | flowering |
| 1 | Aalborg | Undamaged | East Jutland | 57.08 | 9.91 | 5.44 | 56.65 | 11.40 | Meadow | Full Sun | 2020.139 | 0.00 | flowering |
| 2 | Aalborg | Damaged | East Jutland | 57.08 | 9.91 | 5.32 | 76.6 | 11.13 | Meadow | Full Sun | 2430.933 | 0.65 | flowering |
| 2 | Aalborg | Damaged | East Jutland | 57.08 | 9.91 | 5.32 | 76.6 | 11.13 | Meadow | Full Sun | 2160.725 | 0.14 | flowering |
| 3 | Aalborg | Damaged | East Jutland | 57.08 | 9.91 | 4.86 | 133.1 | 12.18 | Meadow | Full Sun | 1098.826 | 0.77 | vegetative |
| 3 | Aalborg | Undamaged | East Jutland | 57.08 | 9.91 | 4.86 | 133.1 | 12.18 | Meadow | Full Sun | 858.85 | 0.00 | vegetative |
| 4 | Falster | Damaged | Islands | 54.80 | 12.09 | 7.66 | 102.8 | 26.34 | Forest | Shade | 2170.537 | 13.64 | immature flowers |
| 4 | Falster | Undamaged | Islands | 54.80 | 12.09 | 7.66 | 102.8 | 26.34 | Forest | Shade | 2189.27 | 0.00 | immature flowers |
| 5 | Falster | Damaged | Islands | 54.80 | 12.09 | 7.93 | 107.2 | 15.80 | Forest | Shade | 2103.608 | 8.31 | immature flowers |
| 5 | Falster | Undamaged | Islands | 54.80 | 12.09 | 7.93 | 107.2 | 15.80 | Forest | Shade | 2457.299 | 0.00 | immature flowers |
| 6 | Falster | Damaged | Islands | 54.80 | 12.09 | 7.89 | 87.9 | 34.32 | Forest | Shade | 3975.556 | 1.39 | immature flowers |
| 6 | Falster | Undamaged | Islands | 54.80 | 12.09 | 7.89 | 87.9 | 34.32 | Forest | Shade | 4547.556 | 0.00 | immature flowers |
| 7 | Grenaa | Damaged | East Jutland | 56.41 | 10.92 | 6.79 | 185.5 | 11.35 | Manicured Park | Full Sun | 1935.134 | 4.86 | flowering |
| 7 | Grenaa | Undamaged | East Jutland | 56.41 | 10.92 | 6.79 | 185.5 | 11.35 | Manicured Park | Full Sun | 1976.337 | 0.00 | flowering |
| 8 | Grenaa | Damaged | East Jutland | 56.41 | 10.92 | 7.02 | 166.1 | 11.10 | Manicured Park | Full Sun | 3591.994 | 1.22 | flowering |
| 8 | Grenaa | Undamaged | East Jutland | 56.41 | 10.92 | 7.02 | 166.1 | 11.10 | Manicured Park | Full Sun | 2342.646 | 0.00 | flowering |
| 9 | Grenaa | Damaged | East Jutland | 56.41 | 10.92 | 6.29 | 129.2 | 9.86 | Manicured Park | Full Sun | 7700.214 | 12.54 | flowering |
| 9 | Grenaa | Undamaged | East Jutland | 56.41 | 10.92 | 6.29 | 129.2 | 9.86 | Manicured Park | Full Sun | 5208.388 | 0.00 | flowering |
| 10 | HannerupSkov | Damaged | East Jutland | 55.59 | 9.72 | 6.2 | 62 | 17.84 | Forest | Shade | 2310.354 | 0.80 | flowering |
| 10 | HannerupSkov | Undamaged | East Jutland | 55.59 | 9.72 | 6.2 | 62 | 17.84 | Forest | Shade | 2849.925 | 0.00 | flowering |
| 11 | HannerupSkov | Damaged | East Jutland | 55.59 | 9.72 | 5.11 | 71.8 | 16.51 | Forest | Shade | 6484.524 | 0.32 | immature flowers |
| 11 | HannerupSkov | Undamaged | East Jutland | 55.59 | 9.72 | 5.11 | 71.8 | 16.51 | Forest | Shade | 7282.472 | 0.00 | immature flowers |
| 12 | HannerupSkov | Damaged | East Jutland | 55.59 | 9.72 | 7.53 | 93.8 | 17.69 | Forest | Shade | 4445.602 | 1.07 | flowering |
| 12 | HannerupSkov | Undamaged | East Jutland | 55.59 | 9.72 | 7.53 | 93.8 | 17.69 | Forest | Shade | 3807.344 | 0.00 | flowering |
| 13 | Langeland | Damaged | Islands | 54.92 | 10.71 | 7 | 112.2 | 12.65 | Agricultural | Full Sun | 3635.21 | 3.32 | immature flowers |
| 13 | Langeland | Undamaged | Islands | 54.92 | 10.71 | 7 | 112.2 | 12.65 | Agricultural | Full Sun | 1835.066 | 0.34 | immature flowers |
| 14 | Langeland | Damaged | Islands | 54.92 | 10.71 | 7.17 | 92.8 | 15.73 | Agricultural | Full Sun | 1255.597 | 2.38 | vegetative |
| 14 | Langeland | Undamaged | Islands | 54.92 | 10.71 | 7.17 | 92.8 | 15.73 | Agricultural | Full Sun | 2741.376 | 0.00 | vegetative |
| 15 | Langeland | Damaged | Islands | 54.92 | 10.71 | 7 | 111.2 | 22.53 | Agricultural | Full Sun | 2147.32 | 7.30 | immature flowers |
| 15 | Langeland | Undamaged | Islands | 54.92 | 10.71 | 7 | 111.2 | 22.53 | Agricultural | Full Sun | 2022.867 | 0.00 | immature flowers |
| 16 | NrNissum | Damaged | West Jutland | 56.55 | 8.42 | 6.65 | 41.9 | 9.72 | Agricultural | Full Sun | 2740.953 | 9.11 | flowering |
| 16 | NrNissum | Undamaged | West Jutland | 56.55 | 8.42 | 6.65 | 41.9 | 9.72 | Agricultural | Full Sun | 2495.906 | 0.00 | flowering |
| 17 | NrNissum | Damaged | West Jutland | 56.55 | 8.42 | 7.71 | 108.7 | 11.33 | Agricultural | Full Sun | 844.488 | 4.54 | flowering |
| 17 | NrNissum | Undamaged | West Jutland | 56.55 | 8.42 | 7.71 | 108.7 | 11.33 | Agricultural | Full Sun | 1132.938 | 0.00 | flowering |
| 18 | NrNissum | Damaged | West Jutland | 56.55 | 8.42 | 6.14 | 138.8 | 14.43 | Agricultural | Full Sun | 1800.361 | 0.75 | flowering |
| 18 | NrNissum | Undamaged | West Jutland | 56.55 | 8.42 | 6.14 | 138.8 | 14.43 | Agricultural | Full Sun | 2247.866 | 0.00 | flowering |
| 19 | Nyraad | Damaged | Islands | 55.01 | 11.96 | 7.56 | 104.5 | 17.06 | Forest | Part Shade | 7409.656 | 5.60 | flowering |
| 19 | Nyraad | Undamaged | Islands | 55.01 | 11.96 | 7.56 | 104.5 | 17.06 | Forest | Part Shade | 5978.531 | 0.00 | flowering |
| 20 | Nyraad | Damaged | Islands | 55.01 | 11.96 | 7.74 | 109.4 | 18.30 | Forest | Part Shade | 1606.262 | 13.73 | immature flowers |
| 20 | Nyraad | Undamaged | Islands | 55.01 | 11.96 | 7.74 | 109.4 | 18.30 | Forest | Part Shade | 3218.295 | 0.00 | immature flowers |
| 21 | Nyraad | Damaged | Islands | 55.01 | 11.96 | 7.73 | 105.8 | 17.66 | Forest | Part Shade | 3183.88 | 16.47 | flowering |
| 21 | Nyraad | Damaged | Islands | 55.01 | 11.96 | 7.73 | 105.8 | 17.66 | Forest | Part Shade | 2543.555 | 1.15 | flowering |
| 22 | Oejsoe | Damaged | East Jutland | 56.29 | 10.61 | 6.07 | 54.4 | 12.68 | Forest | Part Shade | 6514.67 | 1.76 | flowering |
| 22 | Oejsoe | Undamaged | East Jutland | 56.29 | 10.61 | 6.07 | 54.4 | 12.68 | Forest | Part Shade | 6902.549 | 0.00 | flowering |
| 23 | Oejsoe | Damaged | East Jutland | 56.29 | 10.61 | 5.96 | 29.7 | 13.87 | Forest | Part Shade | 3239.084 | 2.49 | flowering |
| 23 | Oejsoe | Undamaged | East Jutland | 56.29 | 10.61 | 5.96 | 29.7 | 13.87 | Forest | Part Shade | 2494.49 | 0.01 | flowering |
| 24 | Oejsoe | Damaged | East Jutland | 56.29 | 10.61 | 4.77 | 61.7 | 13.19 | Forest | Part Shade | 4129.333 | 5.08 | flowering |
| 24 | Oejsoe | Damaged | East Jutland | 56.29 | 10.61 | 4.77 | 61.7 | 13.19 | Forest | Part Shade | 2265.056 | 0.26 | flowering |
| 25 | Randers | Damaged | East Jutland | 56.47 | 10.02 | 7.15 | 72.8 | 13.04 | Manicured Park | Full Sun | 3677.111 | 4.14 | flowering |
| 25 | Randers | Undamaged | East Jutland | 56.47 | 10.02 | 7.15 | 72.8 | 13.04 | Manicured Park | Full Sun | 3915.979 | 0.00 | flowering |
| 26 | Randers | Damaged | East Jutland | 56.47 | 10.02 | 6.92 | 88.7 | 11.81 | Manicured Park | Full Sun | 1345.668 | 0.83 | flowering |
| 26 | Randers | Undamaged | East Jutland | 56.47 | 10.02 | 6.92 | 88.7 | 11.81 | Manicured Park | Full Sun | 1302.758 | 0.00 | flowering |
| 27 | Randers | Damaged | East Jutland | 56.47 | 10.02 | 6.28 | 91.6 | 12.34 | Manicured Park | Full Sun | 1338.75 | 2.52 | flowering |
| 27 | Randers | Undamaged | East Jutland | 56.47 | 10.02 | 6.28 | 91.6 | 12.34 | Manicured Park | Full Sun | 1588.057 | 0.00 | flowering |
| 28 | Ringkoebing | Damaged | West Jutland | 56.10 | 8.23 | 5.83 | 72.3 | 13.96 | Meadow | Full Sun | 4046.962 | 2.20 | flowering |
| 28 | Ringkoebing | Undamaged | West Jutland | 56.10 | 8.23 | 5.83 | 72.3 | 11.93 | Meadow | Full Sun | 3954.658 | 0.00 | flowering |
| 29 | Ringkoebing | Damaged | West Jutland | 56.10 | 8.23 | 5.59 | 60.5 | 11.93 | Meadow | Full Sun | 2230.203 | 1.48 | vegetative |
| 29 | Ringkoebing | Undamaged | West Jutland | 56.10 | 8.23 | 5.59 | 60.5 | 11.93 | Meadow | Full Sun | 1739.432 | 0.00 | vegetative |
| 30 | Ringkoebing | Damaged | West Jutland | 56.10 | 8.23 | 7.92 | 217.5 | 11.19 | Meadow | Full Sun | 1433.442 | 32.31 | flowering |
| 30 | Ringkoebing | Undamaged | West Jutland | 56.10 | 8.23 | 7.92 | 217.5 | 13.96 | Meadow | Full Sun | 1464.245 | 0.13 | flowering |
| 31 | RingkoebingEjstrup | Damaged | West Jutland | 56.18 | 8.28 | 6.32 | 24.8 | 12.12 | Agricultural | Full Sun | 9939.166 | 1.23 | flowering |
| 31 | RingkoebingEjstrup | Undamaged | West Jutland | 56.18 | 8.28 | 6.32 | 24.8 | 12.12 | Agricultural | Full Sun | 7515.323 | 0.00 | flowering |
| 32 | RingkoebingEjstrup | Damaged | West Jutland | 56.18 | 8.28 | 5.92 | 27.1 | 12.03 | Agricultural | Full Sun | 7275.715 | 7.90 | flowering |
| 32 | RingkoebingEjstrup | Undamaged | West Jutland | 56.18 | 8.28 | 5.92 | 27.1 | 12.03 | Agricultural | Full Sun | 7635.522 | 0.00 | flowering |
| 33 | RingkoebingEjstrup | Damaged | West Jutland | 56.18 | 8.28 | 7.89 | 79.5 | 12.33 | Agricultural | Full Sun | 5649.748 | 1.49 | flowering |
| 33 | RingkoebingEjstrup | Undamaged | West Jutland | 56.18 | 8.28 | 7.89 | 79.5 | 12.33 | Agricultural | Full Sun | 4403.146 | 0.00 | flowering |
| 34 | Silkeborg | Damaged | East Jutland | 56.23 | 9.67 | 5.18 | 32.3 | 11.74 | Manicured Park | Full Sun | 3274.088 | 1.51 | flowering |
| 34 | Silkeborg | Undamaged | East Jutland | 56.23 | 9.67 | 5.18 | 32.3 | 11.74 | Manicured Park | Full Sun | 2319.103 | 0.00 | flowering |
| 35 | Silkeborg | Damaged | East Jutland | 56.23 | 9.67 | 5.33 | 33.4 | 12.00 | Manicured Park | Full Sun | 2071.339 | 1.80 | flowering |
| 35 | Silkeborg | Undamaged | East Jutland | 56.23 | 9.67 | 5.33 | 33.4 | 12.00 | Manicured Park | Full Sun | 1824.722 | 0.00 | flowering |
| 36 | Silkeborg | Damaged | East Jutland | 56.23 | 9.67 | 5.46 | 29.7 | 11.68 | Manicured Park | Full Sun | 3284.694 | 21.05 | flowering |
| 36 | Silkeborg | Undamaged | East Jutland | 56.23 | 9.67 | 5.46 | 29.7 | 11.68 | Manicured Park | Full Sun | 3905.996 | 0.00 | flowering |
| 37 | Slaebaek | Damaged | Islands | 55.11 | 10.57 | 7.44 | 115.1 | 18.80 | Forest | Shade | 5965.755 | 0.51 | immature flowers |
| 37 | Slaebaek | Undamaged | Islands | 55.11 | 10.57 | 7.44 | 115.1 | 18.80 | Forest | Shade | 7035.403 | 0.00 | immature flowers |
| 38 | Slaebaek | Damaged | Islands | 55.11 | 10.57 | 6.84 | 277.5 | 15.98 | Forest | Shade | 1180.911 | 6.41 | vegetative |
| 38 | Slaebaek | Undamaged | Islands | 55.11 | 10.57 | 6.84 | 277.5 | 14.05 | Forest | Shade | 3432.232 | 0.00 | vegetative |
| 39 | Slaebaek | Damaged | Islands | 55.11 | 10.57 | 6.45 | 68.4 | 15.98 | Forest | Shade | 1404.738 | 1.69 | vegetative |
| 39 | Slaebaek | Undamaged | Islands | 55.11 | 10.57 | 6.45 | 68.4 | 14.05 | Forest | Shade | 1592.599 | 0.00 | vegetative |
| 40 | Slagelse | Damaged | Islands | 55.43 | 11.46 | 7.22 | 126.8 | 13.40 | Agricultural | Full Sun | 3765.517 | 1.32 | vegetative |
| 40 | Slagelse | Undamaged | Islands | 55.43 | 11.46 | 7.22 | 126.8 | 13.40 | Agricultural | Full Sun | 4039.278 | 0.00 | vegetative |
| 41 | Slagelse | Damaged | Islands | 55.43 | 11.46 | 6.75 | 108 | 13.29 | Agricultural | Full Sun | 5903.454 | 1.38 | immature flowers |
| 41 | Slagelse | Damaged | Islands | 55.43 | 11.46 | 6.75 | 108 | 13.29 | Agricultural | Full Sun | 4903.124 | 0.40 | immature flowers |
| 42 | Slagelse | Damaged | Islands | 55.43 | 11.46 | 5.49 | 103.7 | 12.07 | Agricultural | Full Sun | 5112.955 | 20.03 | flowering |
| 42 | Slagelse | Undamaged | Islands | 55.43 | 11.46 | 5.49 | 103.7 | 12.07 | Agricultural | Full Sun | 5174.492 | 0.00 | flowering |
| 43 | Vissenbjerg | Damaged | Islands | 55.38 | 10.13 | 6.31 | 104.8 | 11.63 | Meadow | Part Shade | 3667.066 | 35.92 | immature flowers |
| 43 | Vissenbjerg | Undamaged | Islands | 55.38 | 10.13 | 6.31 | 104.8 | 11.63 | Meadow | Part Shade | 4573.752 | 0.00 | immature flowers |
| 44 | Vissenbjerg | Damaged | Islands | 55.38 | 10.13 | 7.65 | 110 | 13.16 | Meadow | Part Shade | 2864.567 | 17.45 | immature flowers |
| 44 | Vissenbjerg | Undamaged | Islands | 55.38 | 10.13 | 7.65 | 110 | 13.16 | Meadow | Part Shade | 2515.428 | 0.00 | immature flowers |
| 45 | Vissenbjerg | Damaged | Islands | 55.38 | 10.13 | 7.02 | 401.2 | 12.33 | Meadow | Part Shade | 15100.22 | 0.77 | immature flowers |
| 45 | Vissenbjerg | Undamaged | Islands | 55.38 | 10.13 | 7.02 | 401.2 | 12.33 | Meadow | Part Shade | 9127.273 | 0.00 | immature flowers |
